# Genetic Evidence of an Isolation Barrier between Flea Subspecies of *Citellophilus tesquorum* (Wagner, 1898) (Siphonaptera: Ceratophyllidae)

**DOI:** 10.3390/insects13020126

**Published:** 2022-01-25

**Authors:** Yury Ilinsky, Vasilina Lapshina, Dmitry Verzhutsky, Yulia Fedorova, Sergey Medvedev

**Affiliations:** 1 Laboratory of Molecular Genetics of Insects, Institute of Cytology and Genetics SB RAS, 630090 Novosibirsk, Russia; Lapshina@bionet.nsc.ru (V.L.); i.fedorova8@g.nsu.ru (Y.F.); 2 Center for Mitochondrial Functional Genomics, Immanuel Kant Baltic Federal University, 236041 Kaliningrad, Russia; 3 Irkutsk Antiplague Institute, 664047 Irkutsk, Russia; verzh58@rambler.ru; 4 Parasitology Department, Zoological Institute RAS, 199034 Saint Petersburg, Russia; smedvedev@zin.ru

**Keywords:** *Citellophilus tesquorum*, *COI*, flea, ITS1, ITS2, plague, population, subspecies, *Wolbachia*

## Abstract

**Simple Summary:**

We studied field collections of two flea subspecies that are plague vectors on the vast Palearctic territory. Analysing the molecular–genetic, geographical, morphological, and reproductive isolation criteria, we conclude that these subspecies could be considered different species.

**Abstract:**

This study investigated the relationship between two subspecies of the *Citellophilus tesquorum* flea, *C. t. altaicus* and *C. t. sungaris*, which are vectors of the bacterium *Yersinia pestis* that causes human plague across the vast territories of the Palearctic. Adult fleas were collected from 16 localities and 11 populations in 2019 and 2020. Specimens were morphologically verified for subspecies status and analysed for mitochondrial cytochrome *c* oxidase subunit I (*COI*) DNA, nuclear ribosomal cluster internal transcribed spacer 1 (ITS1) and ITS2, and *Wolbachia*-infection status. Our results demonstrated a genetic difference between *C. t. altaicus* and *C. t. sungaris*. According to mitochondrial data, the genetic distance between clades of *C. t. altaicus* and *C. t. sungaris* was comparable with the species divergence of the genus *Callopsylla*, which is closely related to *Citellophilus*. All studied populations of *C. t. altaicus* were *Wolbachia*-infected, whereas all studied populations of *C. t. sungaris* were symbiont-free. Data for ITS1 and ITS2 had much lower phylogenetic signals than mitochondrial data; however, diagnostic substitutions for *C. t. altaicus* and *C. t. sungaris* delimitation were also revealed. Analysis of a hardly accessible report on cross experiments allowed us to conclude the partial postzygotic isolation between these subspecies. Taken together, the molecular-genetic, geographical, morphological, and reproductive isolation findings verified that *C. t. altaicus* and *C. t. sungaris* subspecies could be considered as different species.

## 1. Introduction

Siphonaptera, commonly known as fleas, comprise a relatively small order of secondarily wingless insects with complete metamorphosis. According to the latest taxonomic revision, this order includes 2005 species and 828 subspecies belonging to 242 genera and 97 subgenera [1].

Fleas are vectors for many pathogens, including *Rickettsia typhi* (which causes endemic typhus), *Rickettsia prowazekii* (rural epidemic typhus) [2], *Bartonella* spp. (cat-scratch disease) [3], and *Yersinia pestis* (plague) [4]. Plague is a dangerous disease, and several hundred cases in humans are reported annually worldwide, predominantly in developing countries [4]. Developed countries have extensive systems for monitoring plague hot-spots that allow preventive actions to be taken to avoid outbreaks. Flea species differ in their ability to transfer plague infection. In total, 257 species are known to be reservoirs of *Y. pestis* [5,6]. *Yersinia pestis* bacteria inhabit the foregut of adult fleas where they form a biofilm that interferes with feeding. While trying to satisfy their hunger, infected fleas actively attack animal or human hosts, and biofilm conglomerates enter the bloodstream transmitting the infection [7].

In previous studies, the focus of researchers has been on the diversity and molecular biology of the pathogenic bacteria, whereas the flea species have been studied much less comprehensively. In terms of population genetic data, the best studied flea species—*Ctenocephalides felis*, *Ctenocephalides canis*, *Pulex irritans*, *Tunga penetrans* and *Xenopsylla cheopis*—are those that are synanotropic. Investigating the diversity of these species has revealed intriguing details of their evolutionary history and candidate cryptic species [8,9,10,11].

Here, we studied populations of *C. tesquorum* (Wagner, 1898) [12], which are active vectors of *Y. pestis* in the vast Palearctic territories [5,13]. This species parasitizes various ground squirrels (*Spermophilus* spp.) in the steppe and mountain regions of Southern Europe, the Caucasus, the Volga region, Central Asia, and Southern Siberia, including Transbaikal and South of Yakutia, Mongolia and Northern regions of China [6]. Nine subspecies of *C. tesquorum* have been described; however, their taxonomic status is under question, because the diagnostic morphological traits vary greatly [14,15,16], and genetic differences between populations in different regions are unknown.

The aim of this study was to clarify the relationship between two broadly distributed subspecies, *C. t. altaicus* and *C. t. sungaris*, which are the main active vectors in several natural plague hot-spots. These subspecies are common in the Altai, Baikalia, Yakutia, Eastern Mongolia and Outer Manchuria territories. In general, *C. t. altaicus* is found in the West, and *C. t. sungaris* is found in the East, and sympatry is noted in some Mongolian regions. Some authors previously defined *C. t. sungaris* as an independent *C. sungaris* species [15,17,18]. This research estimated the isolation between subspecies in order to elucidate their taxonomic status. Adult fleas were collected from populations in the Tuva, Baikalia, Transbaikalia and Yakutia territories. Analysis of the samples compared the mitochondrial DNA (mtDNA) cytochrome *c* oxidase subunit I (*COI*) gene, the internal transcribed spacer 1 (ITS1) and ITS2 regions of the nuclear ribosomal gene, and the *Wolbachia*-infection status, which could be an additional indicator of reproductive isolation between *C. t. altaicus* and *C. t. sungaris* populations.

## 2. Materials and Methods

### 2.1. Sample Collection

**Figure 1:**
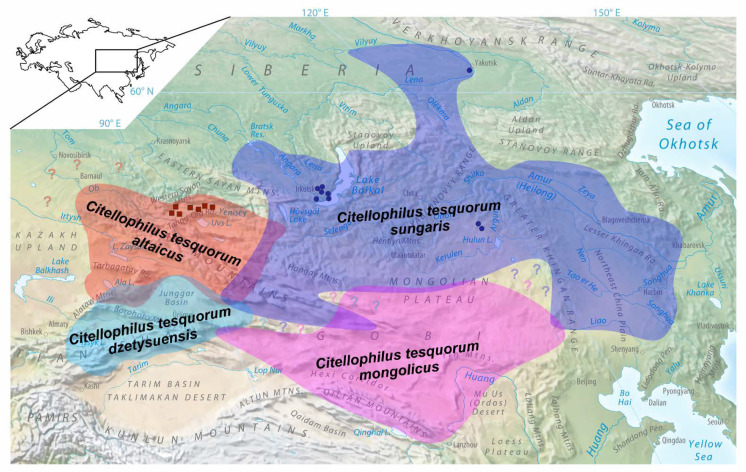
Ranges of four *Citellophilus tesquorum* subspecies. The map based on Tan, Shen [13], and Verzhutsky et al. [19].

**Figure 2:**
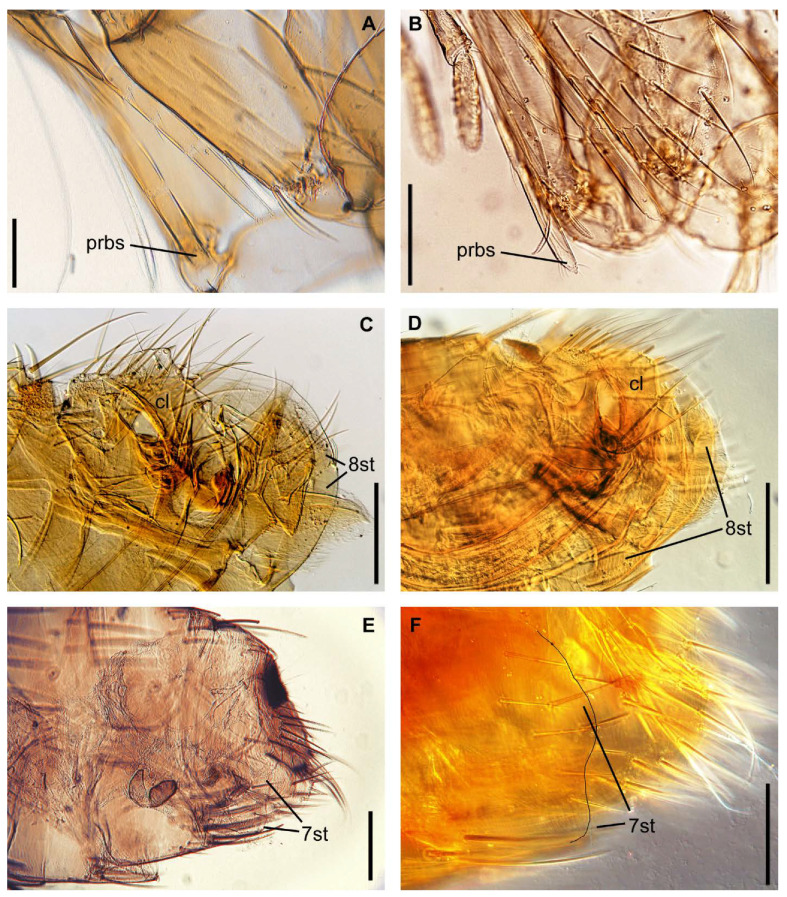
Diagnostic characteristics of *Citellophilus tesquorum* subspecies. Head: ratio of proboscis (prbs) apex to coxa of (**A**) *C. t. altaicus* and (**B**) *C. t. sungaris*. Abdomen: clasper (cl) and sternum VIII (8st) of (**C**) *C. t. altaicus* and (**D**) *C. t. sungaris*. Abdomen: outline of sternum VII (7st) of (**E**) *C. t. altaicus* and (**F**) *C. t. sungaris*. Scale bars = 0.1 mm.

We sampled adult fleas from 16 localities in 2019 and 2020 (Table 1 and Figure 1). Two methods of flea sampling were employed, both of which were conducted according to methodological guidelines MU 3.1.3012-12 of the Federal Centre for State Sanitary and Epidemiological Supervision of Rospotrebnadzor. In method one, ground squirrel nests were excavated, and fleas were collected from the nest substrates. In method two, fleas were collected from the entrances of ground squirrel burrows using a rubber hose with a fleece fabric cover; the hose was pushed several times into the burrow entrance, after which it was examined for fleas. All fleas were stored in ethanol and sent to the Laboratory of Parasitology of the Zoological Institute Russian Academy of Science, Saint Petersburg, Russia, to verify the subspecies status. Details of the subspecies morphological identification are provided in Table 2, Figure 2 and Figure S1. Briefly, three traits of the head and abdomen were analysed to discriminate between *C. t. sungaris* and *C. t. altaicus*, as well as *Citellophilus tesquorum mongolicus* and *Citellophilus tesquorum dzetysuensis*, which were neighbour subspecies to the South (Figure 1).

Before and after morphological verification, the samples were stored in 96% ethanol. The DNA analysis was carried out in the Laboratory of Molecular Genetics of Insects at the Institute of Cytology and Genetics, Novosibirsk, Russia. In total, 61 samples of *C. t. altaicus* and 42 samples of *C. t. sungaris* were used in the molecular study.

### 2.2. DNA Extraction, Amplification and Sequencing

Fleas were individually homogenized in 200 µL of extraction buffer (10 mM TRIS-HCl [pH 8.0], 25 mM ethylenediaminetetraacetic acid (EDTA), 0.5% sodium dodecyl sulphate (SDS), 0.1 M NaCl, and 0.1 mg/mL proteinase K) and incubated for 2 h at 56 °C. The DNA extract was precipitated and diluted in 200 µL of deionised water. One microliter of DNA solution was used for amplification. Three genetic markers were investigated: the nuclear region including ITS1, ITS2, and 5.8S ribosomal RNA (rRNA); the mitochondrial locus *COI*; and the *Wolbachia*-infection status. The mitochondrial locus was amplified with the primer set LCO-1490/HCO-2198 [20]. The nuclear marker was amplified with primers that flanked the 18S and 28S regions as a whole product (2075 base pairs [bp]), or using two overlapping fragments with primers that flanked the 18S and 5.8S regions and the 5.8S and 28S regions; the fragments were sequenced with the primers listed in Table 3. All PCR reactions were performed using BioMaster HS-Taq PCR (2×) (BiolabMix, Novosibirsk, Russia) with a 20 µL volume. The PCR cycling conditions were as follows: initial denaturation 5 min at 95 °C; 35 cycles of denaturing at 95 °C 15 s, annealing at 55 °C for ribosomal DNA and 53 °C for mitochondrial DNA 30 s, elongation at 72 °C 30 s–1 min 30 s depending on the expected amplicon size; and final elongation at 72 °C—3 min. All specimens were examined for *Wolbachia* infection by the nested PCR with the primer set ftsZuniv1/2 for the first round and ftsZf1/r1 for the second round (for details, see [21]). DNA samples of *Drosophila melanogaster* stocks infected with *Wolbachia* and uninfected [22] were used as positive and negative controls. The PCR products were visualised on agarose gel (1.5%) electrophoresis. Amplicons were purified by exonuclease I of *Escherichia coli* (ExoI; New England Biolabs) and sequenced by the BrightDye Terminator Cycle Sequencing kit (Nimagen). Sequences were deposited in the GenBank under accession numbers OL484862–OL484880 for ITS1 and ITS2, and OL504533–OL504557 for *COI*.

### 2.3. Evolutionary Analysis

In addition to our data, we used sequences deposited in the GenBank database by other authors; in particular, we retrieved the following accession numbers: EU770311-14 for the analysis of ITS variation; and MG138174, 77, 78, 80 to 83, 91 and 92 (*Callopsylla* spp.), KM890971, and MF000642 (*C. tesquorum*) for the mtDNA variation. Alignments were generated by the MUSCLE algorithm [29]. DNA polymorphism comprising number of polymorphic sites (S), number of haplotypes (h), haplotype diversity (Hd), nucleotide diversity (Pi) and the fixation index (F_ST_) were calculated using DnaSP v5 [30]. The maximum likelihood (ML) phylogenetic tree for the mtDNA data was reconstructed in Mega6, and the coefficient of differentiation (G_ST_) was calculated [31]. A Templeton, Crandall, and Sing (TCS) gene network [32] was produced by PopArt [33] to represent the genealogical relationships among alleles of nuclear ribosomal genes and their frequencies. An allele of the *Wolbachia ftsZ* locus was checked in the Public Databases for Molecular Typing and Microbial Genome Diversity (PubMLST) [34]. 

## 3. Results

Fleas were collected in 16 localities from 11 populations. The geographical boundaries of the host and flea populations were considered to be the same [35]. Morphological identification of the subspecies status was in agreement with the expectation; five eastern populations were represented by *C. t. sungaris* and six western populations by *C. t. altaicus*. DNA was extracted from 103 fleas: in total, 42 samples were *C. t. sungaris* and 61 were *C. t. altaicus.* All DNA samples were of good quality for PCR analysis, which was checked by amplification with the universal primers LCO-1490/HCO-2190. The full dataset was examined for *Wolbachia*-infection status by nested PCR for bacterial locus *ftsZ*. All populations of *C. t. sungaris* were *Wolbachia*-negative, whereas all populations of *C. t. altaicus* were *Wolbachia*-positive, giving a total of 66% infected samples (Table 1). The analysed *Wolbachia* isolates were characterised by the *ftsZ-56* allele clustered into the A-supergroup (Figure S2), which is common for insects [28].

We sequenced the mitochondrial locus *COI* with the universal primer set of at least one sample for each population. The analysis of alignment (25 samples, 587 bp) revealed values of S = 57 and h = 19; all replacements were synonymous. The values for the Hd and Pi diversity of the entire population were 0.967 and 0.041, respectively. The values of these indices for the subspecies were as follows: for *C. t. sungaris*, S = 18, h = 8, Hd = 0.885 and Pi = 0.013; and for *C. t. altaicus*, S = 25, h = 11, Hd = 0.985 and Pi = 0.014. The F_ST_ and G_ST_ were 0.794 and 0.668, respectively, indicating a high isolation level between subspecies. The *p*-distance between the populations of subspecies was 0.066.

**Figure 3:**
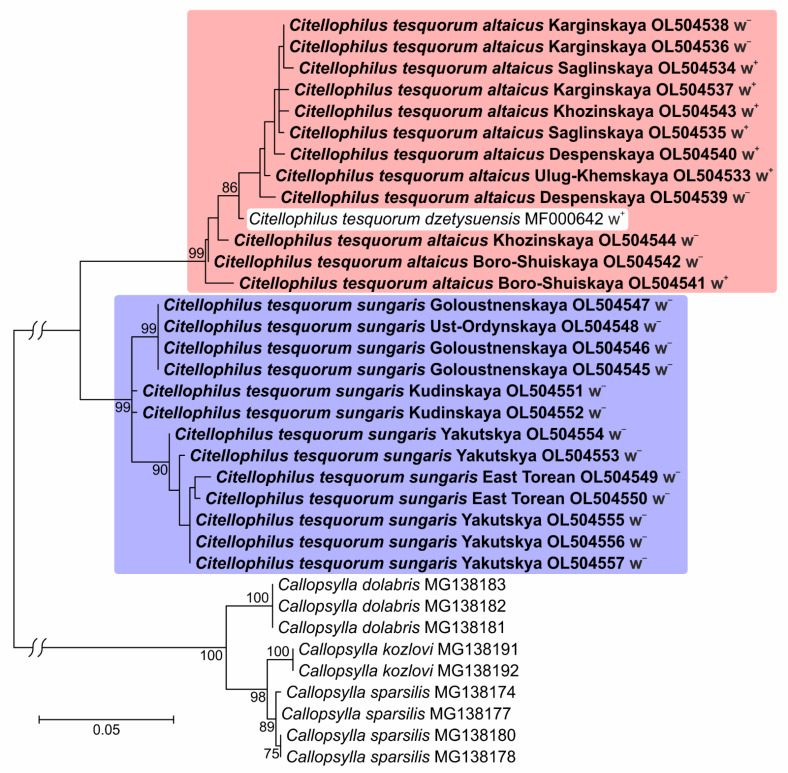
ML-phylogenetic tree of *Citellophilus tesquorum altaicus, C. t. sungaris, C. t. dzetysuensis,* and *Calopsylla* spp. mtDNA based on 587 bp of *COI* sequences (isolates included in this study are shown in bold). GenBank accession numbers, name of flea populations, *Wolbachia*-infection status (w^+^ = infected; w^−^ = uninfected) and bootstrap values (1000 iterations) greater than 70 are provided.

The ML phylogenetic tree of the mtDNA data (Figure 3) had two clades: the first included all *C. t. altaicus* and *C. t. dzetysuensis* retrieved from the GenBank database (MF000642); and the second included only *C. t. sungaris* samples. Therefore, the components of maternal inheritance (mtDNA variation and *Wolbachia* infection) indicated isolation between *C. t. sungaris* and *C. t. altaicus*.

**Figure 4:**
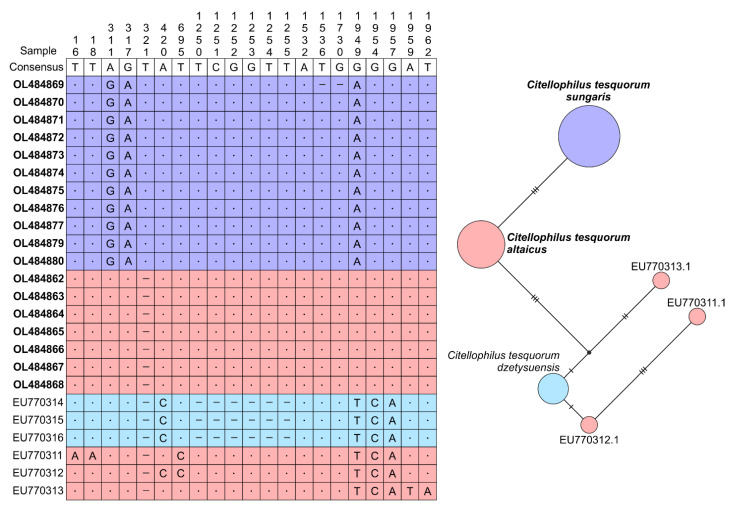
A—Nucleotide polymorphisms of ITS1 and ITS2 for *Citellophilus tesquorum altaicus, C. t. sungaris* and *C. t. dzetysuensis* (highlighted in accordance with Figure 1). Bold GenBank numbers indicate those included in this study. The number of polymorphic sites is indicated after excluding ambiguous positions from the alignment. B—Relationships (TCS network) of rRNA alleles reconstructed by PopArt.

To estimate the differentiation between subspecies according to nuclear genes, we sequenced the ITS1 and ITS2 and located 5.8S rRNA locus between them. As with the mtDNA variation analysis, we aimed to obtain sequences from all populations. However, ITS1 and ITS2 variation was very low, so we sequenced only 19 samples. Despite the low variation, the observed polymorphism subdivided the *C. t. altaicus* and *C. t. sungaris* samples. The length of the nuclear sequences was in the range of 1999 to 2001 bp. Five sites were characterised by an ambiguous signal that could be explained by errors in sequencing or by heterozygosity, so they were excluded from the analysis. Three isolates of *C. t. altaicus* and three of *C. t. dzetysuensis* were retrieved from the GenBank and added to the analysis. The final alignment of 25 samples comprised 1968 bp (Figure 4) and was characterised by the following parameters: S = 12, h = 6, Hd = 0.700 and Pi = 0.00176. The F_ST_ and G_ST_ values between the samples of *C. t. altaicus* and *C. t. sungaris* were 1.0, demonstrating complete isolation between the subspecies (see the Supplementary Material).

## 4. Discussion

Traditional morphological analyses combined with molecular-genetic investigation is a powerful approach to clarify relationships at the species level. In previous studies of fleas, notable results were obtained for *Ct. felis* whereby the subspecies *Ct. f. orientalis* was reclassified as the species *Ct. orientalis* [8,9], and *Ct. f. felis* and *Ct. f. strongylus* were found to be synonymous [36]. Based on a conflict of genetics and phenotype variation, a wide morphological plasticity was found in females of the genus *Ctenophthalmus* [37]. Moreover, two cryptic lineages (species) were identified within *Pulex irritans* [11].

Our results clearly demonstrated genetic differences between *C. t. altaicus* and *C. t. sungaris*. The most evident divergence of these subspecies was observed in their mtDNA data. The genetic distance between the clades of *C. t. altaicus* and *C. t. sungaris* was comparable to the species divergence within the *Callopsylla* genus, which is closely related to *Citellophilus*. In addition, the sequences of *C. t. dzetysuensis* and *C. t. mongolicus* derived by other authors clustered into the *C. t. altaicus* clade (Figure S3). The data for ITS1 and ITS2 showed much weaker phylogenetic signals. Only three replacements and one *indel* were found per 2 Kbp; however, they were diagnostic for *C. t. altaicus* and *C. t. sungaris* divergence. Additional isolates of *C. t. altaicus* and *C. t. dzetysuensis* deposited in GenBank showed even greater divergence from *C. t. sungaris* (Figure 4). Finally, the populations of *C. t. sungaris* differed from *C. t. altaicus* in *Wolbachia*-infection status. The symbiont was found in all populations of *C. t. altaicus,* and *Wolbachia* infection was found in *C. t. dzetysuensis* (MF045776–MF045779 and MF04583–MF045786). Notably, *Wolbachia* infection is often associated with flea diversity as summarized by Yudina et al. [38]. The sequence of the *Wolbachia ftsZ* gene isolated from *C. t. altaicus* in our isolates was clustered into the A-supergroup. The analysis of the *wsp* locus of *Wolbachia* isolated from *C. t. dzetysuensis* revealed two types of symbionts clustered also into the A-supergroup (Figure S2).

A crucial element of species discrimination is reproductive isolation. We analysed a hardly accessible report on reciprocal crosses of *C. t. altaicus* and *C. t. sungaris* [39] (see Supplementary Materials). In both cross directions, the number of F_1_ progeny per female was in the range of 1.2–6.4, whereas in the control crosses (within subspecies) it was 45.4–51.0. Low fertility (2.0–5.4) was also observed in the F_2_ progeny. This indicated incomplete postzygotic isolation between the subspecies. The authors noted that the hybrids were slightly larger than the parents, demonstrating higher rates of fluctuating asymmetry and morphoses. The hybrids were also tested to produce a biofilm of *Y. pestis* and to infect laboratory animals [40]. Both parameters were higher in the progeny than in the parent subspecies. These features could decrease hybrid fitness in the field via more effective infection of the host population and increased starvation levels due to *Y. pestis* infection.

Taken together, the molecular–genetic, geographical, morphological and reproductive isolation data indicate that the *C. t. altaicus* and *C. t. sungaris* are long-term isolated and could be considered different species. Even from a sceptical perspective, the subspecies features clearly indicate a case of incomplete speciation. Here, it is important to mention that Jordan [17], Cyprich et al. [18] and Lewis [15] provided *C. t. sungaris* as the independent species *C. sungaris*. 

The next step for our research will be to clarify the relationships among the other subspecies of *C. tesquorum*. Preliminary results based on the limited sequences retrieved from the GenBank database indicate that *C. t. mongolicus* and *C. t. dzetysuensis* do not show significant differences from *C. t. altaicus.* However, genetic data on Eastern European and Caucasian populations are lacking to date. 

## Figures and Tables

**Table 1:** Characteristics of data collection and *Wolbachia* infection.

Subspecies of *Citellophilus tesquorum*	Population	No of Localities	No of Samples	No of *Wolbachia* Infected Samples
*sungaris*	Goloustnenskaya	3	7	0
*sungaris*	Ust-Ordynskaya	2	7	0
*sungaris*	East Torean	2	19	0
*sungaris*	Kudinskaya	1	4	0
*sungaris*	Yakutskaya	1	5	0
*altaicus*	Ulug-Khemskaya	1	15	15
*altaicus*	Saglinskaya	1	9	7
*altaicus*	Karginskaya	2	20	9
*altaicus*	Despenskaya	1	6	3
*altaicus*	Boro-Shaiskaya	1	6	3
*altaicus*	Chozinskaya	1	5	3

**Table 2:** Morphological delimitation traits of four *Citellophilus tesquorum* subspecies.

Trait	*C. t. altaicus*	*C. t. sungaris*	*C. t. mongolicus*	*C. t. dzetysuensis*
Head: ratio of proboscis apex to coxa and trochanter	reach apex of coxa, or middle of trochanter (Figure 2A)	reach apex of trochanter (Figure 2B)	reach middle or apex of trochanter	reach middle of trochanter
Abdomen: presence of membranous appendage of sternum VIII apical part	present (Figure 2C and Figure S1A)	absent (Figure 2D and Figure S1C)	absent (Figure S1D)	absent (Figure S1B)
Abdomen: presence of lateral sinus of posterior margin sternum VIII	absent (Figure 2E and Figure S1E)	present or absent (Figure 2F and Figure S1G)	present (Figure S1H)	no data (Figure S1F)

**Table 3:** Primers used in the study.

Primer	Target	5′-3′ Sequence	Reference
ITS5-f1	ribosomal region	GGAAGTAAAAGTCGTAACAAGG	[23]
ITS2-r2	ribosomal region	CAAGGTTTCCGTAGGTGAACCTG	[24]
ITS1ctf2	ribosomal region	CGCGTACAGGCAGATTATCA	this study
ITS1ctr2	ribosomal region	GCCCGCACTCAAACATTAAA	this study
ITS1ctf	ribosomal region	CGTGCTTCGGTGTGTGTTTT	this study
ITS1ctr	ribosomal region	GGACAAATTCGCTCTCACGC	this study
ITS2-f2	ribosomal region	GGGTCGATGAAGAACGCAGC	[25]
ITS1-r1	ribosomal region	GCTGCGTTCTTCATCGACCC	[26]
ITS2-f3	ribosomal region	GACCACTCCTGGCTGAGG	this study
ITS1-r2	ribosomal region	CCAGGAGTGGTCCGGGAACAGTATC	this study
28S-r2	ribosomal region	TAGTTTCTTTTCCTCCGCTTAA	this study
28S-r1	ribosomal region	GCCGCTACTAAGGGAATCCTA	this study
HCO-2198	*COI*, mitochondrial gene	TAAACTTCAGGGTGACCAAAAAATCA	[20]
LCO-1490	*COI*, mitochondrial gene	GGTCAACAAATCATAAAGATATTGG	[20]
ftsZuniv1	*Wolbachia* symbiont	GG(CT)AA(AG)GGTGC(AG)GCAGAAGA	[27]
ftsZuniv2	*Wolbachia* symbiont	ATC(AG)AT(AG)CCAGTTGCAAG	[27]
ftsZf1	*Wolbachia* symbiont	ATYATGGARCATATAAARGATAG	[28]
ftsZr1	*Wolbachia* symbiont	TCRAGYAATGGATTRGATAT	[28]

## Data Availability

The data presented in this study are available in article or Supplementary Materials.

## Supplementary Materials

The following are available online at https://www.mdpi.com/article/10.3390/insects13020126/s1, S1. Pdf file of the article: Nikitin A.Ya., Nechaeva L.K. Hybridization of flea subspecies as a possible basis for the method of regulating the number of vectors. //Organization of plague surveillance and prevention measures. Mater. Interstate Scientific and Practical Conference—Alma-Ata (in Russian).—1992. 3. 400–403; S2. Pdf file of the article: Nikitin A.Ya., Bazanova L.P., Nechaeva L.K., Korzun V.M., Khabarov A.V., Kozets L.I. Experimental study of the ability of hybrids bred from two subspecies of the flea *Citellophilus tesquorum* to transmit plague bacillus. //Medical Parasitology and Parasitic Diseases (in Russian).—1995. 4. 15–17; Figure S1. Diagnostic features of *Citellophilus tesquorum* subspecies; Figure S2. Phylogenetic relationships of *ftsZ* and *wsp Wolbachia* allels; Figure S3. Phylogenetic tree of a 404-bp region of the *COI* gene reconstructed by the maximum likelihood method based on the T92 + I model. All bootstrap (100 iterations) values are indicated.
