# Supplementary material for: Genetic Evidence of an Isolation Barrier between Flea Subspecies of Citellophilus tesquorum (Wagner, 1898) (Siphonaptera: Ceratophyllidae)
Source: Insects. 2022 Jan 25;13(2):126. doi: 10.3390/insects13020126 (PMC8879408; doi:10.3390/insects13020126)
Supplement: Supplementary file 1 [file insects-13-00126-s001.zip › Figures S1¿CS3.pdf]

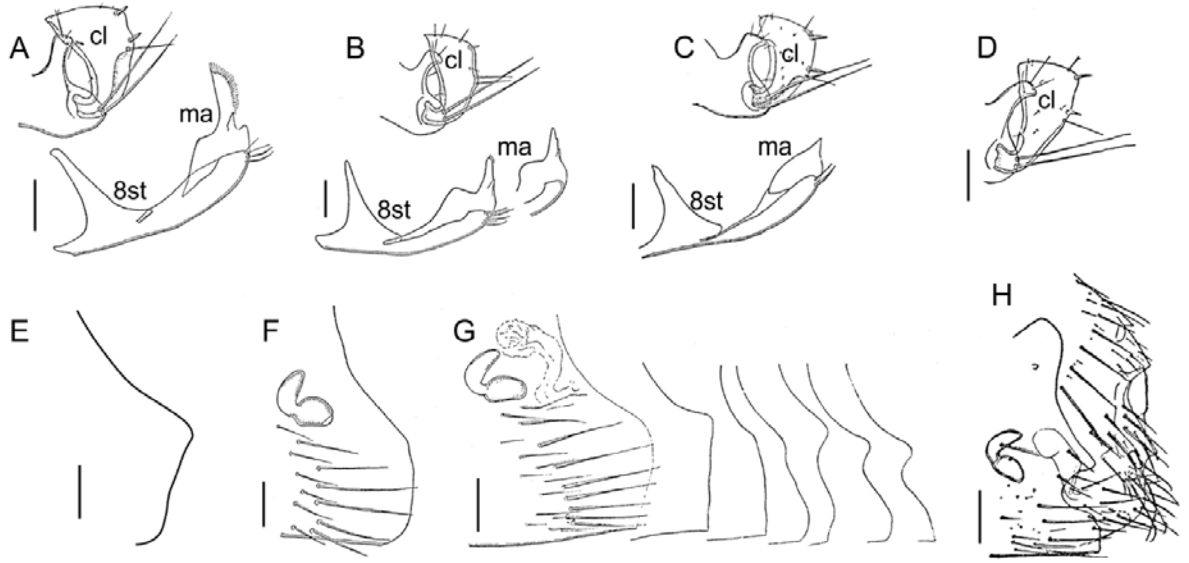

Figure S1. Diagnostic characteristics of *Citellophilus tesquorum* subspecies. Abdomen: clasper (cl), membranous appendage (ma) and sternum VIII (8st) (**A**) *C. t. altaicus* (from: Ioff *et al.*, 1965, fig. 196), (**B**) *C. t. dzetysuensis* (from: Ioff *et al.*, 1965, fig. 197), (**C**) *C. t. sungaris* (from: Ioff *et* Scalon, 1954, fig. 121), (**D**) *C. t. mongolicus* (from: Ioff *et* Scalon, 1954, fig. 122). Abdomen: sternum VII (**E**) *C. t. altaicus*, (**F**) *C. t. dzetysuensis* (from: Ioff *et al.*, 1965, fig. 199), (**G**) *C. t. sungaris* with modifications (from: Ioff *et* Scalon, 1954, fig. 123), (**H**) *C. t. mongolicus* (from: Goncharov *et al.*,1989, fig. 75). Scale bars = 0.1 mm.

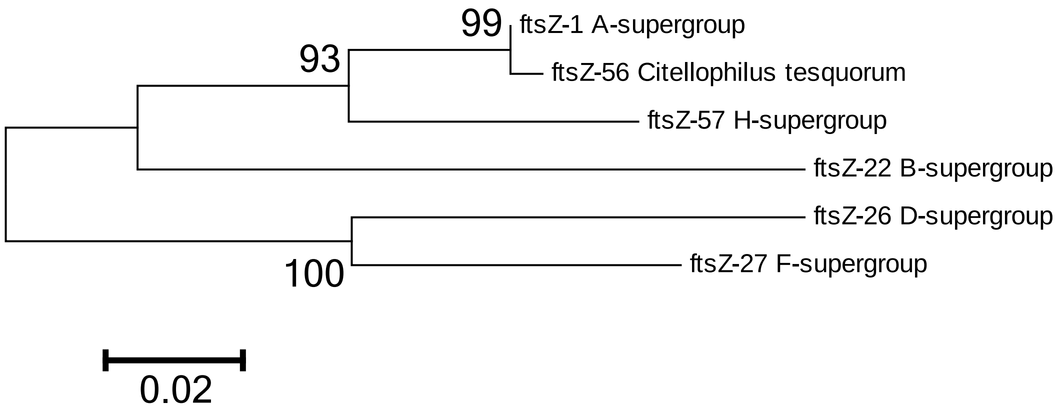

(A)

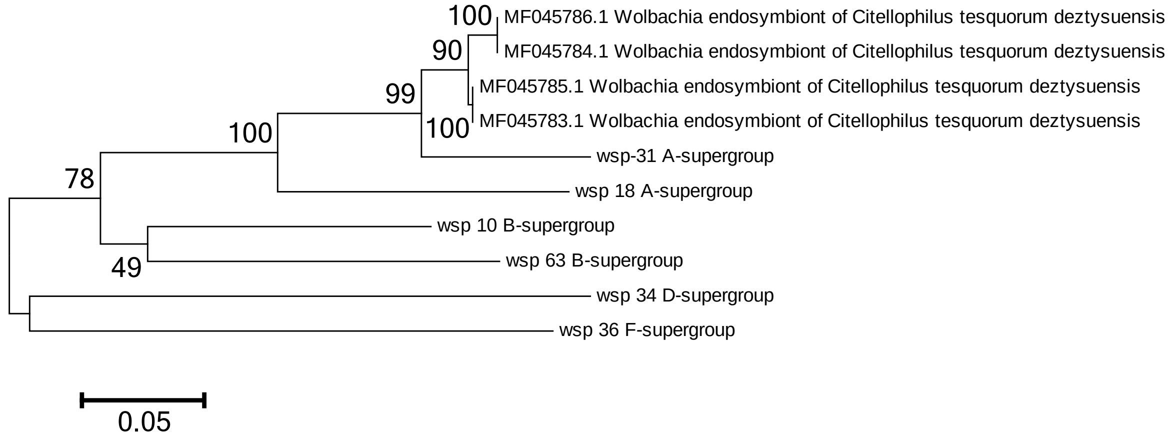

(B)

Figure S2. (**A**)Maximum likelihood phylogenetic tree of *ftsZ* alleles of *Wolbachia* symbionts was reconstructed with Mega6 [Tamura et al., 2013]. The *Wolbachia* isolate of *Citellophilus tesquorum* belong to supergroup A. (**B**) Maximum likelihood phylogenetic tree of *wsp* alleles of *Wolbachia* symbionts was reconstructed with Mega6 [Tamura et al., 2013]. The *Wolbachia* isolates of *Citellophilus tesquorum deztysuensis* are clustered into supergroup A.

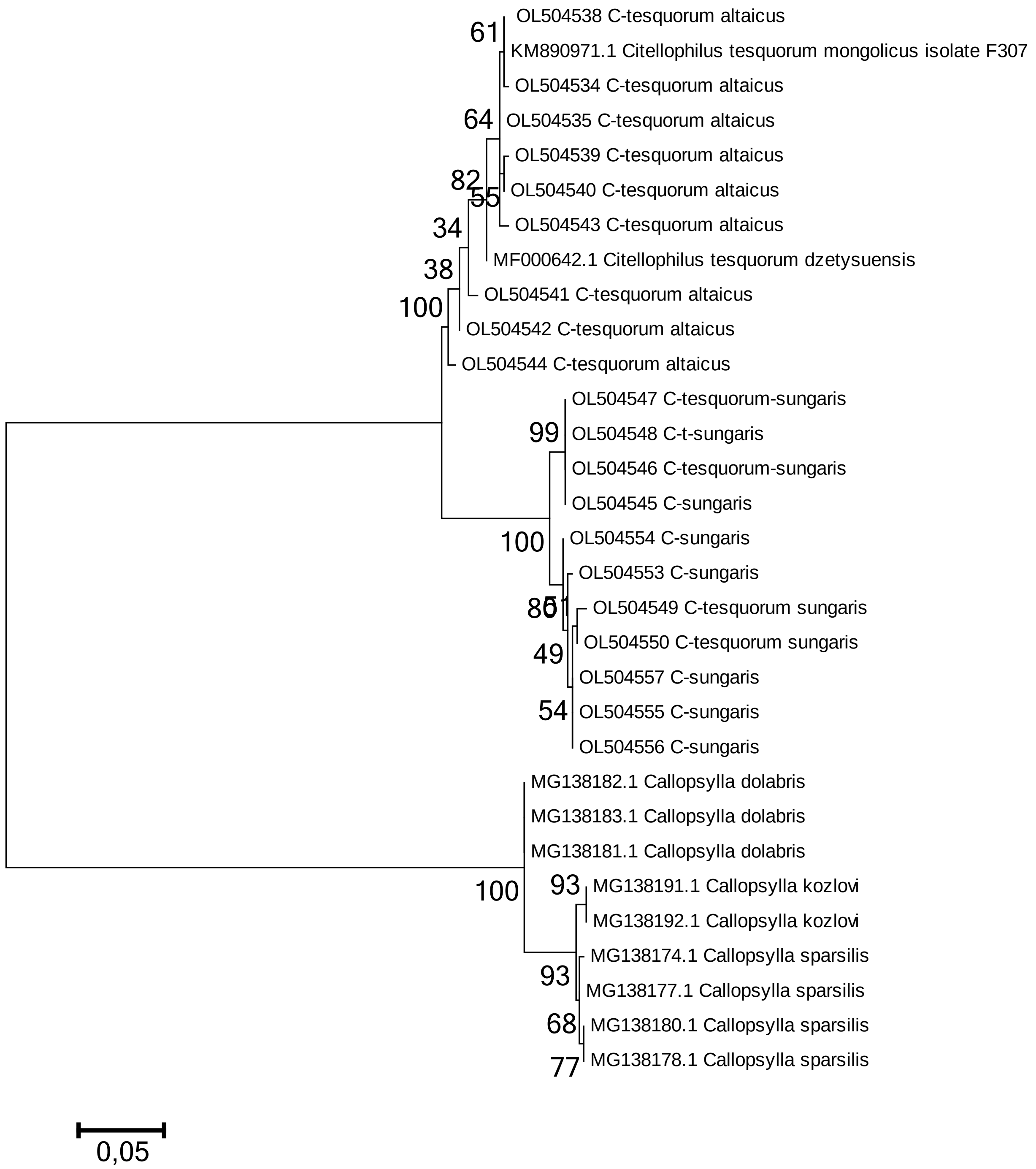

Figure S3. Molecular phylogenetic tree of 404 bp part of COI gene is reconstructed by Maximum Likelihood method based on the T92+I model. All bootstrap (100 iterations) values are indicated.

Иофф И.Г., Скалон О.И. 1954. Определитель блох Восточной Сибири, Дальнего Востока и прилегающих районов. М., Медгиз, 276 с. [Ioff I.G., Skalon O.I. 1954. Guide of fleas of Eastern Siberia, the Far East and neighboring areas. Moscow, Medgiz Publishing, 276 pp. (in Russian)].

Иофф И.Г., Микулин М.А., Скалон О.И. 1965. Определитель блох Средней Азии и Казахстана. М., Медицина, 371 с. [Ioff I.G., Mikulin M.A., Skalon O.I. 1965. Guide of fleas of Central Asia and Kazakhstan. Moscow, Medicina Publishing, 371 pp. (in Russian)].

Гончаров А.И., Ромашева Т.П., Котти Б.И., Баваасан А., Жигмид С. 1989. Определитель блох Монгольской Народной Республики. Улан-Батор, 417 с. [Goncharov A.I., Romasheva T.P., Kotti B.I., Bavaasan A., Zhigmid S. 1989. Guide of fleas of Mongolia. Ulan-Bator, 417 pp. (in Russian)].

Tamura, K., Stecher, G., Peterson, D., Filipski, A., Kumar, S., 2013. MEGA6: molecular evolutionary genetics analysis version 6.0. Mol. Biol. Evol. 30, 2725–2729.
