# Supplementary material for: Genetic Evidence of an Isolation Barrier between Flea Subspecies of Citellophilus tesquorum (Wagner, 1898) (Siphonaptera: Ceratophyllidae)
Source: Insects. 2022 Jan 25;13(2):126. doi: 10.3390/insects13020126 (PMC8879408; doi:10.3390/insects13020126)
Supplement: Supplementary file 1 [file insects-13-00126-s001.zip › S1-1992_Nikitin_Nechaeva_ocr.pdf]

А-Я 1992, т.3

А. Я. Никитин, Л. К. Нечаева

ГИБРИДИЗАЦИЯ ПОДВИДОВ БЛОХ КАК ВОЗМОЖНАЯ ОСНОВА МЕТОДА РЕГУЛЯЦИИ ЧИСЛЕННОСТИ ПЕРЕНОСЧИКА

Иркутск

До настоящего времени химические методы являются доминирующими в борьбе с блохами в природных очагах чумы. Однако в связи с наличием определенных отрицательных последствий действия инсектицидов на окружающую среду неослабевает интерес исследователей к разработке более "экологичных" препаратов и методов борьбы на принципиально новой основе.

Способ борьбы с вредными насекомыми путем их стерилизации достаточно хорошо обоснован на уровне теоретических моделей, проверен в лабораторных экспериментах и в ряде случаев был успешно использован на практике (Книплинг,1971; Рукавишников,1979).

На наш взгляд можно, выделить минимум три подхода к регуляции численности насекомых путем их стерилизации: 1 - хемостерилянтами воздействуют непосредственно на естественную популяцию насекомых; 2 - в лабораторных условиях химическими или физическими методами вызывают стерильность обычно самцов, с последующим внедрением их в "регулируемую" популяцию; 3 - используют интродукцию линий, рас, дающих после скрещивания с особями "регулируемой" популяции бесплодное потомство. Все три метода, обычно применяют после химической дезинсекции для уничтожения выжившей части популяции или продлении достигнутой эффективности проведенных мероприятий. Наиболее "экологически" чистым и в большинстве случаев экономически оправданным будет третий подход. Вместе с тем, именно он пока является наименее разработанным.

В двух из трех сибирских очагов чумы основным переносчиком

возбудителя является Citellophyllus tesquorum (Голубинский, Жовтый, Лемишева, 1987). По некоторым данным и в третьем природном очаге этому виду принадлежит значительная роль в эпизоотиях чумы (Машковский, 1987). Вместе с тем, показано (Голубинский, Жовтый, Лемишева, 1987), что в разных очагах обитают два разных подвида этих блох, причем оба хорошо разводятся в инсектарных условиях (Жовтый, Нечаева, 1983). Следовательно, теоретически имеется хороший плацдарм для разработки метода регуляции численности блох за счет интродукции в естественные популяции самцов несвойственных данному очагу подвида насекомых в расчете на малую численность и плохую приспособленность потомков от подобного рода гибридизации.

Для проверки этого предположения в 1990-91гг. в инсектарии Иркутского противочумного института проведены реципрокные скрещивания лабораторных культур C. t. altaicus и C. t. sungaris. Сразу же отметим, что гибридизация проходит успешно, хотя численность потомков, приходящихся на одну самку, резко падает (табл. 1). Тем самым подтверждается таксономический статус этих переносчиков.

В табл. 1 приведены некоторые данные, характеризующие уровень приспособленности первого и второго поколения гибридов и родительских особей. Полученные результаты не позволяют говорить о наличии различий в сроках прохождения отдельных стадий онтогенеза. Вместе с тем, в проведенных скрещиваниях подвидов наблюдается уменьшение числа потомков по сравнению с обоими исходными формами. Если выразить этот эффект через формулу Чемберлена, используемую для оценки эффективности химической стерилизации, то получим величину, равную приблизительно 90 %, что на 11 % выше, чем эффективность стерилизации самцов этого вида в лабораторных опытах одним из наиболее мощных для блох на сегодняшний день хемостерилянтом тиоТЭФ (Чумакова,1974).

Таблица 1

Анализ некоторых компонент приспособленности у двух подвидов C.tesquorum (C.t.a. - altaicus; C.t.s. - sungaris) и гибридов от реципрокных скрещиваний между ними

N п.п.		Вариант скрещивания	Время выплода первых личинок по отдельным опытам (сутки)	Минимальные сроки метаморфоза (сутки)	Число потомков от 1 самки за время наблюдений	Вес 10 самок в мг	Среднее число овариол у 1 самки
1		C.t.a.*C.t.a.	19	45	51.0	7	6.4
2		C.t.s.*C.t.s.	13	45	45.4	8	10
3	F1	C.t.a.*C.t.s.	28; 11; 18	43; 40	3.8; 6.3	4	6.6
4	F1	C.t.s.*C.t.a.	28; 13	43; 40	1.2; 4.4	5	6.1
5	F2	3 F1 * 3 F1	12; 17	33	2.0	-	-
6	F2	4 F1 * 4 F1	12; 17	29	5.4	-	-

Таблица 2

Сравнение мерных признаков и уровня их изменчивости у родительских подвидов и их гибридов

Полусумма по вариантам	Относительная длина						Уровень изменчивости признаков (σ)					
	головы		бедра		голени		головы		бедра		голени	
	♀	♂	♀	♂	♀	♂	♀	♂	♀	♂	♀	♂
Родители	609.6	536.0	570.8	524.7	555.0	510.3	22.09	20.00	24.09	21.24	39.08	22.42
F1	620.0	545.0	596.2	532.4	575.1	508.3	14.74	6.24	26.46	12.09	16.11	13.82
F2	588.6	522.9	563.7	508.4	551.7	489.2	32.26	1994	39.78	28.19	39.95	28.57

Анализ трех мерных признаков (длина головы, бедра и голени третьей пары ног) позволяет сделать следующие предварительные выводы (табл. 2): гибриды первого поколения несколько крупнее, чем полусумма соответствующих размеров их родителей, т.е. наблюдается некоторое доминирование более крупного подвида или проявляется эффект гетерозиса; изменчивость особей первого поколения по совокупности изученных признаков в целом ниже, чем у родителей и гибридов второго поколения; есть некоторые данные, говорящие о том, что у гибридов увеличивается уровень флуктуирующей асимметрии и возрастает частота морфозов.

В заключение хотелось бы подчеркнуть такую немаловажную деталь: рассматриваемый биологический метод борьбы с блохами не только может помочь уменьшить антропогенную нагрузку на среду, но также и привести к получению дополнительной эффективности мероприятий по оздоровлению очага. Во-первых, эволюционно не отшлифованные гибриды могут оказаться неприспособленными к жизни в данных условиях. Во-вторых, если мы считаем, что паразитарная триада является продуктом коэволюции ее элементов, то очень вероятно, что у гибридов может нарушиться способность взаимодействовать с возбудителем и хозяином (носителем инфекции). Причем, из двух событий: усилинея или ослабления способности к блокообразованию и передаче возбудителя более вероятным является второе, поскольку любое вмешательство в эволюционно сложившиеся механизмы скорее поведет к нарушению их нормальной работы. Очевидно, при этом мы понимаем всю опасность подобных экспериментов над природой и необходимость тщательного лабораторного анализа соответствия ожидаемых и получаемых результатов.
