# Supplementary material for: Genetic Evidence of an Isolation Barrier between Flea Subspecies of Citellophilus tesquorum (Wagner, 1898) (Siphonaptera: Ceratophyllidae)
Source: Insects. 2022 Jan 25;13(2):126. doi: 10.3390/insects13020126 (PMC8879408; doi:10.3390/insects13020126)
Supplement: Supplementary file 1 [file insects-13-00126-s001.zip › S2-1995_Nikitin_et_al_ocr.pdf]

микробов в виде конгломератов имеют большее значение в проявлении вирулентности чумного микроба для лабораторных животных, чем цепочки бактерий чумы и одиночные микробы, что является одним из важных факторов в механизме трансмиссивной передачи возбудителя.

ЛИТЕРАТУРА

1. *Акиев А.К.* // Природно-очаговые инфекции и их профилактика. — Саратов, 1991. — С. 118 — 122.
2. *Брюханова Г.Д., Акиев А.К., Бейер А.П., Михайлова В.К.* // Эпидемиология; микробиология и иммунология бактериальных и вирусных инфекций. — Ростов-н/Д., 1989. — С. 13 — 15.
3. *Ващенок В.С.* Блохи (Siphonaptera) — переносчики возбудителей болезней человека и животных. — Л., 1988.
4. *Жалко-Титаренко В.П., Григорьев А.В., Бондаренко В.М., Ковальчук В.К.* // Журн. микробиол. — 1988. — № 9. — С. 28 — 32.
5. *Классовский Л.Н., Бибикова В.А.* // Там же. — 1968. — № 4. — С. 118 — 121.
6. *Мерков А.М., Поляков Л.Е.* Санитарная статистика (Пособие для врачей). — Л., 1974.
7. Методические указания по определению чувствительности микроорганизмов к антибиотикам методом диффузии в агар с использованием дисков. — М., 1983.
8. *Пшеничнов В.А., Семенов Б.Ф., Зезеров Е.Г.* Стандартизация методов вирусологических исследований. — М., 1974.
9. *Русакова Л.В., Новокрещенова Н.С., Загнибородова Е.Н.* и др. // Проблемы особо опасных инфекций. — Саратов, 1976. — Вып. 6 (52). — С. 5 — 12.
10. Учебно-методическое пособие по лабораторной диагностике и изучению биологических свойств чумного микроба и возбудителей некоторых других природно-очаговых инфекций. — Иркутск, 1972.

Поступила 26.04.95

PLAGUE BACILLUS: THE STATE OF AGGREGATION

G.D.Bryukhanova, A.K.Akiyev, A.P.Beyer

Hemoglobin enhances the aggregation of the plague bacillus in fluids (p > 0.99), the proportion of its action on this process being 46.7 %. The aggregated forms of the plague bacillus as conglomerates are of greater value in the virulence of the plague bacillus for white mice than are a series of plague bacilli or single microbes, which is an important factor in the mechanism of bacillus transmission. Block is an aggregated status of the plague bacillus in the gizzard of an effective vector.

© Коллектив авторов, 1995

А.Я. Никитин, Л.П. Базанова, Л.К. Нечаева, В.М. Корзун, А.В. Хабаров, Л.И. Козец

ЭКСПЕРИМЕНТАЛЬНОЕ ИЗУЧЕНИЕ СПОСОБНОСТИ ГИБРИДОВ ОТ СКРЕЩИВАНИЯ БЛОХИ CITELLOPHILLUS TESQUORUM ДВУХ ПОДВИДОВ ПЕРЕДАВАТЬ ВОЗБУДИТЕЛЯ ЧУМЫ

Иркутский противочумный институт Сибири и Дальнего Востока, Госкомсанэпиднадзор России

В одном природном очаге чумы могут обитать блохи, относящиеся к разным популяциям, подвидам и видам — переносчикам *Yersinia pestis* [3, 4, 6, 9 — 12]. С учетом известных фактов возможной гибридизации [8, 12, 17] отдельных видов и подвидов блох, представляет интерес исследование роли этого процесса, являющегося мощным фактором физиологической перестройки организма [7] в формировании общей эпизоотической картины в очаге чумы. В частности, в исследовании, проведенном на клещах [14], показано, что на стыке ареалов тех подвидов клещей, для которых установлена возможность гибридизации, наблюдаемая картина эпизоотий может быть достаточно «непривычной».

Целью данной работы явилось рассмотрение способности вирулентных штаммов *Y.pestis* формировать блок преджелудка у гибридов от скрещивания двух подвидов блохи *C.tesquorum* и возможности участия гибридных насекомых в передаче возбудителя носителю. Априори представлялись вероятными три варианта взаимодействия организма гибридов и возбудителя: от неспособности микробных клеток интенсивно размножаться и, соответственно, блокировать преджелудок насекомых в силу отсутствия естественной коэволюции сочленов этой системы до нормальной (то есть сходной с родительскими подвидами) или возросшей эффективности трансмиссии возбудителя.

Материал и методы. В трех проведенных опытах использованы гибридные блохи 1, 3 и 4-го поколений (F1, F3, F4), происходящие от скрещивания особей из инсектарных культур двух подвидов: C.t.altaicus, доминирующего вида в Тувинском природном очаге чумы, и C.t.sungaris, основного переносчика в Забайкальском очаге [6]. Из литературы известно, что ареалы этих подвидов сближаются в Монголии на Северном Хангае [12].

В опыте с F1 для заражения блох

Таблица 1

Формирование блока преджелудка у гибридов и родительских подвидов при заражении разными штаммами чумного микроба

Вариант опыта	Штамм И-3266			Штамм И-1966		
	начальная зараженность (%)	число блох в группе	% блох с блоком	начальная зараженность (%)	число блох в группе	% блох с блоком
F1	100	114	6,1±2,25	100	124	8,9±2,56
C.t.altaicus	100	112	12,5±3,12	80	110	8,2±2,62
C.t.sungaris	100	83	7,2±2,84	100	116	6,9±2,35

использовано два вирулентных штамма *Y.pestis*: И-1996 и И-3266 (LD_{50} соответственно составляет 20 и 10 микробных клеток для белых мышей), выделенных в Забайкальском и Тувинском природных очагах. В опытах с F3 и F4 использован только штамм И-3266.

Имаго F1 получены в результате скрещивания самок C.t.sungaris и самцов C.t.altaicus , а F3 и F4 — из культуры, происходящей от реципрокного скрещивания. В каждом опыте для сравнения исследовали и родительские подвиды. Для экспериментов брали молодых неразмножавшихся особей.

Блох заражали через биомембрану. Концентрация бактерий заражающей смеси равнялась 100 млн. м.к./мл. Инфицированных насекомых содержали при температуре 20 — 23°С и влажности 80 — 96%. Через 2 — 3 суток блох подкармливали на белых мышах. После каждой подкормки отделяли особи, у которых сформировался блок преджелудка. Грызунов, после снятия с них блох, оставляли для дальнейших наблюдений. В каждом варианте проведено не менее 7 подкормок блох. Павших мышей, а также проживших свыше 25 суток после подкормки на них блох, исследовали паталогоанатомическим и бактериологическим методами.

Статистическая обработка материала проведена методами анализа качественных признаков [15].

Результаты и обсуждение. Начальная инфицированность блох в опытах составила от 75 до 100% (у гибридов во всех опытах 100%). Полученные данные показывают, что у гибридных блох, также как и у родительских подвидов, формируется блок преджелудка и они способны к трансмиссии возбудителя белым мышам (табл. 1 — 3).

Доля блокированных гибридов колеблется в опытах в достаточно широких пределах (от 6,1 до 33,3%). Первые блоки формируются после 2 -4-й подкормки. Специальные наблюдения показали, что блокированные гибриды заражают мышей при их подкормках в составе небольших групп (1 — 3 особи). Достоверных различий по частоте передачи возбудителя и по доле блокированных блох, в зависимости от использованного штамма микроба, не наблюдали ни в одном из вариантов опыта с F1 (табл. 1, 2). Исследование павших мышей показало типичную при заболевании чумой патолого-анатомическую картину. Определенных различий в среднем времени гибели мышей после заражающего кормления (табл. 2), а также в характере и степени поражения органов между вариантами опытов при визуальном анализе не обнаружено.

Блохи F3 и F4 передают возбудителя несколько эффективнее родительских подвидов. Кроме того, у них чаще формируется блок преджелудка (табл. 3),

Таблица 2

Трансмиссия чумного микроба гибридами и родительскими подвидами блох при их заражении разными штаммами возбудителя

Вариант опыта	Штамм И-3266			Штамма И-1966		
	Число мышей в опыте		Среднее время гибели (дни)	Число мышей в опыте		Среднее время гибели (дни)
	всего	павших		всего	павших	
F1	10	4	5±0,41	10	7	5±0,75
C.t.altaicus	10	5	5±0,77	10	4	4,3±0,48
C.t.sungaris	10	2	5,5±0,50	10	3	4,7±0,67

Таблица 3

Трансмиссия чумного микроба гибридами и родительскими подвидами блох

Вариант опыта	Начальная зараженность (%)	Число блох в группе	% блох с блоком	Число мышей в опыте	
				всего	павших
F3	100	90	33,3±4,97	7	3
C.t.altaicus	100	126	4±1,75	8	0
C.t.sungaris	93	231	3,5±1,21	23	0
F4	100	251	8,4±1,75	9	5
C.t.altaicus	100	120	1,7±1,18	9	1
C.t.sungaris	75	128	5,5±2,02	7	2

особенно в F3. В частности, последний показатель у гибридов (33%) не только статистически достоверно выше, чем у родительских подвидов в этом же опыте, но и превосходит все известные из литературы [1 — 3, 5, 6] максимальные значения блокообразования (8 — 12%), характерные для данных подвидов блох. В целом, по трем опытам, гибриды заразили 19 из 36 мышей (52,8%), C.t.altaicus — 10 из 37 (27%), C.t.sungaris — 7 из 50 (14%). Полученные результаты, как и обнаруженные ранее межпопуляционные различия по трансмиссивной способности блох у одного и того же вида [9, 10, 16], могут указывать на роль генотипических особенностей особей исследуемых культур в определении эффективности трансмиссии возбудителя.

Таким образом, в гибридных особях, также как и в насекомых родительских подвидов, микроб может интенсивно размножаться, образуя глыбки и формируя блок преджелудка. Гибридизация генетически достаточно сильно дивергировавших блох не нарушает их способности передавать возбудителя чумы, и в некоторых случаях усиливает этот процесс. Такой феномен позволяет предположить возможность активизации эпизоотического процесса в местах гибридизации. В перспективе особого внимания заслуживает вопрос о последствиях гибридизации особей из отдельных популяций и субпопуляционных структур, в силу: 1) доказанной сложной пространственной структуры населения блох в очаге [3, 4, 11]; 2) большей вероятности их гибридизации по сравнению с блохами, относящимися к разным подвидам и видам; 3) уже известных значительных различий в эпизоотической значимости внутрипопуляционных структур [3]. Гибридизация как метод увеличения наследственного разнообразия может стать инструментом для исследования характера генетической детерминации различных признаков у блох.

ЛИТЕРАТУРА

1. *Базанова Л.П.* Эпизоотологическое значение блохи Citellophilus tesquorum altaicus в Тувинском природном очаге чумы: Автореф. дис. ... канд. биол. наук. — Саратов, 1993.
2. *Базанова Л.П., Жовтый И.Ф., Маевский М.П.* и др.//Мед. паразитол. — 1991. — № 1. — С. 24 — 26.
3. *Вержуцкий Д.Б.* Пространственная структура населения массовых видов блох длиннохвостого суслика в Тувинском природном очаге чумы и ее эпизоотологическое значение: Автореф. дис. ... канд. биол. наук. — Саратов, 1990.
4. *Вержуцкий Д.Б.*//Конференция по проблеме: «Современные аспекты природной очаговости, эпидемиологии и профилактики особо опасных инфекционных болезней»: Тезисы докладов. — Ставрополь, 1993. — С. 26 — 28.
5. *Воронова Г.А., Феоктистов А.З.*//Проблемы особо опасных инфекций. — Саратов, 1979. — Вып. 4. — С. 50 — 53.
6. *Голубинский Е.П., Жовтый И.Ф., Лемешева Л.Б.* О чуме в Сибири. — Иркутск, 1987. — С. 135 — 174.
7. *Захаров В.М.* Асимметрия животных (популяционно-феногенетический подход). — М., 1987. — С. 62 — 117.
8. *Золотова С.И., Ихсанова З.А.*//Научная конф. противочумных учреждений Средней Азии и Казахстана, 10-я: Тезисы докладов. — Алма-Ата, 1979. — С. 99 — 101.
9. *Князева Т.В.* Экология блох — переносчиков чумы в Прикаспийском северо-западном природном очаге и их эпидемиологическое значение: Автореф. дис. ... канд. биол. наук. — Саратов, 1987.
10. *Князева Т.В., Топорков В.П., Бережнов А.З.* и др.// Труды противочумных учреждений СССР. — Саратов, 1989. — С. 116 — 121.
11. *Козлов М.П., Чумаков И.В.*//Всесоюзная научн.-практ. конф. по проблеме: «Актуальные вопросы эпиднадзора в природных очагах чумы. Природная очаговость чумы в высокогорьях»: Тезисы докладов. — Ставрополь, 1985. — С. 147 — 149.
12. *Лабунец Н.Ф.*//Изв. Иркутск. противочумного ин-та. — 1968. — Т. 27. — С. 231 — 240.
13. *Никитин А.Я., Нечаева Л.К.*//Межгосударственная науч.-практ. конф. по проблеме: «Организация эпиднадзора по чуме и меры ее профилактики»: Тезисы докладов. — Алма-Ата, 1992. — С. 400 — 403.
14. *Поспелова-Шторм М.В.*//Мед. паразитол. — 1950. — № 6. — С. 514 — 519.
15. *Рокицкий П.Ф.* Биологическая статистика. — Минск, 1967. — С. 179 — 198.
16. *Сержанов О.С., Хрусцелевская Н.М., Чумаченко В.Д.* и др.//Проблемы особо опасных инфекций. — Саратов, 1979. — Вып. 4. — С. 58 — 60.
17. *Якунин Б.М., Куницкая Н.Т.*//Паразитология. — 1992. — Т. 26, вып. 5. — С. 418 — 423.

Поступила 03.04.95

EXPERIMENTAL STUDY OF THE ABILITY OF HYBRIDS BRED FROM TWO SUBSPECIES OF THE FLEA CITELLOPHILUS TESQUORUM TO TRANSMIT PLAGUE BACILLUS

A.Ya. Nikitin, L.P. Bazanova, L.K. Nechayeva, V.M. Korzun, A.V. Khabarov, L.I. Kozets

The ability to transmit of plague bacillus was studied in the first-, third— and fourth-generation hybrids bred from C.t.altaicus and C.t.sungaris. The fleas were inoculated with two virulent strains from the Tuva and Transbaikal natural foci of plague. The bacillus was demonstrated to infect the hybrids, form gizzard block, and be transmitted to white mice, causing the animals' death.

© Коллектив авторов, 1995

А.В.Наумов, И.А.Кузьмиченко, Т.М.Тараненко, С.А.Коровкин, Г.В.Ермакова

БИОХИМИЧЕСКИЕ АСПЕКТЫ ПАТОГЕННОСТИ ВОЗБУДИТЕЛЯ ЧУМЫ

Российский научно-исследовательский противочумный институт «Микроб», Саратов

Достижения в области молекулярной природы патогенности позволили охарактеризовать и сгруппировать биомолекулы, вовлеченные в развитие инфекционного процесса, по функциональному признаку в соответствии с той ролью, которую они выполняют во взаимодействии микроба с клетками макроорганизма [18]. Важнейшей начальной стадией этого взаимодействия является биологическое узнавание и специфическая адгезия бактериальной клетки, способствующая колонизации возбудителем соответствующей ткани. Другую группу факторов патогенности составляют продукты, препятствующие процессу узнавания микроба фагоцитарными клетками и позволяющие популяции выжить и размножиться. Биомолекулы с токсической функцией нарушают регуляторные механизмы эукариотической клетки и вызывают ее специфическое поражение.

У чумного микроба физико-химическое состояние поверхности имеет своеобразное строение, с большим числом белков и наличием обнаженных липидных частей ЛПС, придающих гидрофобность и выполняющих защитную функцию [17]. В формировании высокой энергии поверхности и сильного отрицательного заряда значительный вклад принадлежит продуктам плазмид, особенно pPst. На первичной фазе адгезии происходит процесс лиганд-рецепторного узнавания за счет высокой специфичности адгезинов, морфологически являющихся пилями (фимбриями), синтезируемыми при 37 °С и низком значении рН и обладающими лектиноподобным действием. Дезинтеграция высокомолекулярной структуры нативных пилей ультразвуком приводит к утрате лектиноподобных свойств при сохранении иммунохимической активности. Имеются данные в пользу того, что сигнал «37 °С — низкий рН» имитирует условия в фаголизосомах макрофагов, где происходит размножение фагоцитированных клеток чумного микроба, при этом система «пили — клеточный рецептор» индуцирует в клетке-мишени целый комплекс биохимических изменений [2, 12].

Сопоставление имеющихся данных позволяет считать белок пилей (пилин), рН 6-антиген и белок J (антиген 4) аналогичными компонентами чумного микроба [37, 38]. Об этом свидетельствует общность их основных свойств — зависимость биосинтеза при 37 °С от рН, гемагглютинирующая активность, обеспечение прикрепления микроба к клеткам-мишеням, цитотоксичность, нарушение переваривающей функции макрофагов, близкая молекулярная масса и, наконец, их существенное участие в экспрессии вирулентности возбудителя чумы.

Важным звеном при клеточном контакте является у возбудителя чумы и действие нейраминидазы [42]. Десиалирование эритроцитов нейраминидазой усиливает их связывание с пилями адгезии, а у лейкоцитов белых мышей снижает уровень хемилюминесцинции под влиянием пилей. Нейраминидаза цитотоксична для перитонеальных макрофагов белых мышей и морских свинок, после контакта с ней лейкоциты реагируют на капсульный антиген резким увеличением продукции перекиси водорода [5]. Все это свидетельствует о ее многофункциональной природе, которая очевидно шире, чем обеспече-

Page 4/4
